# Disentangling direct and pleiotropic SNP effects in alfalfa (*Medicago sativa* L.) using causal graph learning

**DOI:** 10.1038/s41598-026-35876-w

**Published:** 2026-01-14

**Authors:** Yangming Lee, Cesar A. Medina, Zhanyou Xu

**Affiliations:** 1 RoCAL, Rochester Institute of Technology, Rochester, NY 14623 United States; 2 Department of Agronomy and Plant Genetics, University of Minnesota, Saint Paul, MN 55455 United States; 3 Agricultural Research Service, Urbana, IL 61801 United States; 4 Department of Crop Sciences, The University of Illinois Urbana-Champaign, Urbana, USA

**Keywords:** Alfalfa, Causal graphical framework, Direct parent SNPs, Upstream Hub SNPs, Causal discovery, Computational biology and bioinformatics, Genetics, Plant sciences

## Abstract

Alfalfa (*Medicago sativa* L.) is a critical forage crop whose improvement depends on resolving the complex genetic architecture of agronomic traits. While genome-wide association studies (GWAS) effectively identify statistically associated markers, they often fail to distinguish direct genetic effectors from indirect or pleiotropic signals arising from linkage disequilibrium and population structure. Here, we present a causal graph based genomic discovery framework that integrates de-confounded feature screening with causal graph learning to infer directional dependency structures from observational genomic data. Using Double Machine Learning to control for confounding and the PC algorithm for structural learning, we construct directed acyclic graphs that distinguish *Direct Parent SNPs (DPSs)*, representing local effectors within the Markov Blanket of a trait, from *Upstream Hub SNPs (UHSs)*, representing pleiotropic regulators with broad network connectivity. Applied to four stem-related traits in alfalfa, the framework reduces genome-wide associations to compact, interpretable causal-consistent networks. Predictive validation demonstrates that DPSs consistently outperform both upstream UHSs and random controls, confirming their role as precise trait-specific biomarkers, while UHSs exhibit limited direct predictive power consistent with signal dilution along causal pathways. Together, these results demonstrate that causal graph learning can act as a biologically grounded regularizer for GWAS in polyploid crops, enabling principled marker prioritization and providing a structural foundation for future multi-omics integration.

## Introduction

Alfalfa (*Medicago sativa* L.) is a cornerstone forage crop, with over 14.8 million acres harvested in 2022 in the U.S., valued at over $12.9 billion^1^. As a premier feedstock for the dairy industry, it supports high-quality milk production while contributing vital ecosystem services, including soil health improvement and biological nitrogen fixation^2^. Consequently, enhancing agronomic traits such as biomass yield, stem fiber digestibility, and winter hardiness remains a priority for sustainable agriculture^3,4^.

Genome-wide association studies (GWAS) have served as the primary tool for dissecting the genetic architecture of these traits^5^. By testing for statistical dependencies between phenotypes and single nucleotide polymorphisms (SNPs), GWAS has successfully cataloged markers associated with yield^6^, digestibility, and stress resistance^7,8^. Advanced methods, such as mixed linear models (MLMs), have further refined this process by correcting for population structure and kinship^9^.

However, the autotetraploid nature of alfalfa presents unique challenges. The complex genome structure, extensive linkage disequilibrium (LD), and high heterozygosity create a “fog of correlation” where non-causal markers frequently show strong statistical association due to physical linkage or population stratification^10–12^. Crucially, traditional GWAS remains fundamentally associative. It identifies genomic regions correlated with a trait but cannot resolve impact directions. It struggles to distinguish whether a marker is a functional driver, a downstream proxy, or a spurious correlate induced by a latent confounder. Without resolving these directional dependencies, breeders are often left with a list of redundant markers rather than a map of the underlying genetic mechanism.

To capture the non-linear epistatic interactions missed by linear GWAS models, Machine Learning (ML) approaches, such as Random Forest (RF)^13^ and Support Vector Machines (SVM), have been widely adopted as fundamental tools^7^. Yet, while these “black-box” models significantly improve predictive accuracy, they lack mechanistic interpretability. To address this, network-based approaches have been developed. While graph representation learning has successfully identified associations in other biological domains, such as RNA-disease networks^14,15^, these methods often focus on prediction rather than resolving directional causality. More advanced causal inference frameworks, including Structural Equation Modeling (SEM)^16,17^ and QTL-directed dependency graphs^18,19^, have been explored, but often rely on strong prior assumptions^20^.

To bridge the gap between statistical association and biological causation, this work introduces a Causal-Informed Genomic Discovery Framework. Moving beyond simple correlation, we integrate de-confounded feature screening (Double Machine Learning) with structural learning (PC Algorithm) to construct Directed Acyclic Graphs (DAGs) from observational genomic data.

The primary contributions of this framework include:

1. **Resolution of Genetic Architecture:** Unlike standard GWAS, our framework resolves the topological structure of the genome, distinguishing between **DPSs** (local effectors forming the Markov Blanket) and **UHSs** (pleiotropic regulators).
2. **De-confounded Signal Detection:** By integrating Double Machine Learning, we rigorously filter out spurious associations driven by population stratification, isolating markers with robust, direct causal effects.
3. **Reduction of False Discoveries:** We demonstrate that causal graph construction acts as a biological regularizer, pruning indirect edges driven by LD chains and significantly reducing the false discovery rate compared to linear baselines.
4. **Foundation for Systems Breeding:** By successfully identifying key drivers and resolving impact directions at the SNP level, this work provides a validated structural scaffold. This enables breeders to prioritize “Direct Parent” SNPs (DPSs) for precise genomic prediction while identifying “Upstream Hub” SNPs for investigating broader regulatory networks.

## Materials and methods

### Plant materials and experimental design

The plant materials utilized in this study originated from two cycles of recurrent selection for stem fiber digestibility, as detailed by^21^. The study panel consisted of 500 alfalfa genotypes derived from five populations: the base unselected population (Cycle 0, C0; a mixture of six commercial varieties) and four advanced populations (C1 and C2) selected for high and low *in vitro* neutral detergent fiber digestibility (IVNDFD) at 16-h and 96-h intervals.

The panel was established in a field trial at the University of Minnesota Agricultural Experiment Station (Saint Paul, MN). The experiment employed a Randomized Complete Block Design (RCBD) with three replicates per genotype (1,500 plots total). To account for field micro-variation, plots were arranged in a contiguous spatial array (50 rows $\times$ 30 columns).

### Phenotypic data acquisition and modeling

Four agronomic traits were evaluated during the 2022 and 2023 growing seasons. To ensure consistency with the genomic analysis, trait names follow the nomenclature used in the dataset:

- **Stem Color and Stem Fill:** Scored as binary traits (0 = Yellow/Hollow, 1 = Brown/Solid).
- **Stem Strength (Winter Standability):** Scored on an ordinal scale (1–5), representing plant lodging resistance after snow cover (1 = $<10\%$ erect, 5 = $>70\%$ erect). This trait is referred to as stem_strength in the analysis.
- **Winter Injury:** Scored on an ordinal scale (1–5), where 1 indicated plant death and 5 indicated vigorous, uninjured growth^7^.

To minimize environmental noise, phenotypic values were modeled using ASReml-R. We applied a linear mixed model incorporating spatial coordinates (row and column) to control for spatial heterogeneity. Best Linear Unbiased Predictions (BLUPs) were estimated for the genotype effect (*Y*), which served as the phenotype input for subsequent causal discovery. Although Stem Color and Stem Fill were recorded as binary categories, they were modeled as quantitative traits to generate continuous BLUPs reflecting the underlying genetic propensity.

### Genomic data and quality control

Leaf tissue from individual plants was sampled for genotyping using the Alfalfa DArTag panel, targeting approximately 3,000 SNP loci distributed genome-wide^7,22^.

Given the autotetraploid nature of alfalfa ($2n=4x=32$), genotypic calls were processed to capture allele dosage. Markers were encoded as numeric values $\{0, 1, 2, 3, 4\}$ representing the count of the alternative allele. To ensure statistical robustness, the raw genotypic matrix underwent quality control filtering:

1. **Call Rate:** Markers with $>10\%$ missing data were removed.
2. **Minor Allele Frequency (MAF):** Markers with MAF $< 0.05$ were excluded to prevent spurious associations driven by rare variants.

The final high-dimensional dataset comprised $N=500$ genotypes and $M \approx 2,434$ high-quality SNP markers. This numeric dosage matrix formed the basis for the “quasi-continuous” assumption utilized in the subsequent structural learning steps.

## Methodology: causal-informed genomic discovery framework

### The core framework: from statistical association to causal DAGs

Our primary objective is to transcend associative GWAS by reconstructing the underlying Causal Directed Acyclic Graph (DAG) governing phenotypic variation in autotetraploid Alfalfa. We model the genomic system as a DAG, $\mathcal {G} = (V, E)$, where nodes *V* represent genetic markers (SNPs) and the phenotype, and directed edges *E* represent direct influences unmediated by other observed variables.

This transition from statistical association to causal structure relies on a fundamental distinction:

- **Statistical Significance** (derived from P-values) merely rejects the null hypothesis of independence.
- **Causal Significance** (derived from structural learning) implies a mechanism where intervention on the parent node (SNP) alters the probability distribution of the child node (Trait).

To bridge this gap, we employ the PC Algorithm^23^. Our choice of this constraint-based method is grounded in its rigorous treatment of Conditional Independence (CI), which biologically maps to the “screening-off” property of Linkage Disequilibrium (LD): a true causal variant renders its linked non-causal proxies conditionally independent of the trait ($SNP_{proxy} \perp \!\!\perp Trait \mid SNP_{causal}$).

### The dimensionality challenge and causal-consistent screening

A fundamental challenge in genomic causal discovery is the “Curse of Dimensionality” ($p \approx 3000 \gg n = 500$ in this work)^24,25^. The PC algorithm is computationally intractable on the full genome, and global confounders induce dense spurious edges that violate the sparsity assumption of causal DAGs^26^.

To address this, we implement a Causal-Consistent Feature Screening step using Double Machine Learning (DoubleML), as explained below^27^.

#### Why partially linear regression (PLR)?

We explicitly reject standard screening methods (e.g., Pearson correlation, standard Lasso) because they are inherently associative and prone to “regularization bias.” Standard Lasso may shrink the coefficient of a causal SNP to zero if it is highly correlated with a confounder. In contrast, we employ the Partially Linear Regression (PLR) model. This model isolates the linear causal effect $\theta$ by orthogonalizing the SNP and the Trait against the confounding vector *X*:

1 $$\begin{aligned} Y = \theta D + g(X) + \zeta , \quad D = m(X) + \nu \end{aligned}$$

By solving for $\theta$ using the residuals ($Y - \hat{g}(X)$ and $D - \hat{m}(X)$), DoubleML recovers the direct structural parameter $\theta$, free from the influence of *X*.

#### The role of PCA: Proxy for population and environment

We define the confounding vector *X* as the top 10 Principal Components (PCs) of the genotype matrix^28^. In our framework, PCs serve a dual purpose as high-dimensional proxies:

1. **Population Stratification:** PCs capture the systematic variance due to lineage and kinship, preventing spurious associations driven by family structure.
2. **Environmental Confounding:** Since geographic origin strongly correlates with genetic background in Alfalfa (e.g., cold-tolerant varieties in northern latitudes), PCs implicitly capture unobserved environmental confounders (e.g., temperature, photoperiod) that systematically affect both genotype distribution and phenotypic expression.

#### Biological assumption: linearity vs. complexity

For the nuisance functions *g*(*X*) and *m*(*X*), we employed LassoCV (linear)^29^ rather than non-linear learners^13,30^. This decision is grounded in the genetic architecture of autotetraploids:

- **Additive Main Effects:** While non-linear effects (dominance and epistasis) exist, additive effects constitute the primary component of heritable variance.
- **Robustness:** Given the limited sample size ($n = 500$), attempting to model complex non-linear interactions risks severe overfitting. A robust linear approximation captures the “Main Effect” ($D \rightarrow Y$) reliably, which is the primary target for breeding selection.

### Structural learning via the PC algorithm

With the candidate nodes ($V_{cand}$) identified, we proceed to resolve the “edges” (causal paths) using the PC Algorithm^23^.

#### Why constraint-based learning?

We select the PC algorithm over score-based methods (e.g., GES) due to its reliance on Conditional Independence (CI) tests. In the context of genomics, CI directly maps to the biological concept of LD pruning but in a causal direction:

- **Scenario:** If $SNP_A$ and $SNP_B$ are in high LD and both associate with the trait.
- **Resolution:** The PC algorithm tests: $SNP_A \perp \!\!\perp Trait \mid SNP_B$. If this holds, the edge $SNP_A \rightarrow Trait$ is removed, correctly identifying $SNP_B$ as the proximal cause (or “Hub”) that screens off $SNP_A$.

#### Graph construction and pruning

The algorithm is initialized with a complete undirected graph over $V_{cand} \cup \{Trait\}$.

1. **Skeleton Discovery:** We perform hierarchical independence tests using Fisher’s Z-transform at significance level $\alpha =0.05$. This parameter was tuned to balance sparsity with connectivity; stricter thresholds were found to fragment biological pathways. Although genotypic data in autotetraploids is discrete ($0, 1, \dots , 4$), treating allele dosage as a **quasi-continuous variable** is a standard approximation in quantitative genetics^31^.
2. **Orientation:** V-structures (colliders, $X \rightarrow Z \leftarrow Y$) are oriented based on the separation sets found in the previous step.
3. **Topological Pruning and Directional Constraints:** To distinguish relevant mechanisms from side-associations and ensure biological plausibility, we extracted the **Ancestral Subgraph** of the phenotype. This step acts as a filter for “reverse causality.” Since a phenotype cannot biologically alter a germline genotype, any edges directed from the Trait to a SNP ($\text {Trait} \rightarrow \text {SNP}$) represent statistical artifacts (e.g., selection bias or feedback loops) rather than biological mechanisms. By defining the final graph $G_{final}$ as the set of all nodes *V* such that there exists a directed path $V \rightarrow \dots \rightarrow \text {Trait}$, we effectively enforce a “taboo” on reverse edges. This guarantees that the final network topology respects the directionality of the central dogma ($\text {Genotype} \rightarrow \text {Phenotype}$) and retains only the upstream causal drivers.

### Implementation and robustness protocols

#### Screening stability (statistical validation)

To ensure that our candidates possess both statistical and causal validity, we implemented **Stability Selection**:

- We executed the DoubleML screening $N_{rep}=100$ times with 5-fold cross-fitting.
- Only SNPs consistently identified as significant ($P < 10^{-4}$) across random splits were admitted to the graph. This rigorous threshold filters out noise, retaining only those loci with a stable, de-confounded signal.

#### Structural learning configuration

- **Independence Testing:** We utilized Fisher’s Z-test ($\alpha =0.05$) to assess conditional independence.
- **Topological Pruning:** Post-discovery, we extracted the Ancestral Subgraph of the phenotype. Nodes lacking a directed path to the trait in the final CPDAG were pruned, ensuring the final network represents only the causal ancestry of the target trait.

## Results

We applied our Causal-Informed Discovery framework to four agronomic traits in autotetraploid Alfalfa: Stem Color, Stem Fill, Stem Strength, and Winter Injury. To demonstrate the topological resolution of our pipeline, we present a detailed structural analysis of Stem Color as a representative case study. Comparative analyses of shared genetic mechanisms across all traits are presented in subsequent sections.

**Fig. 1:**
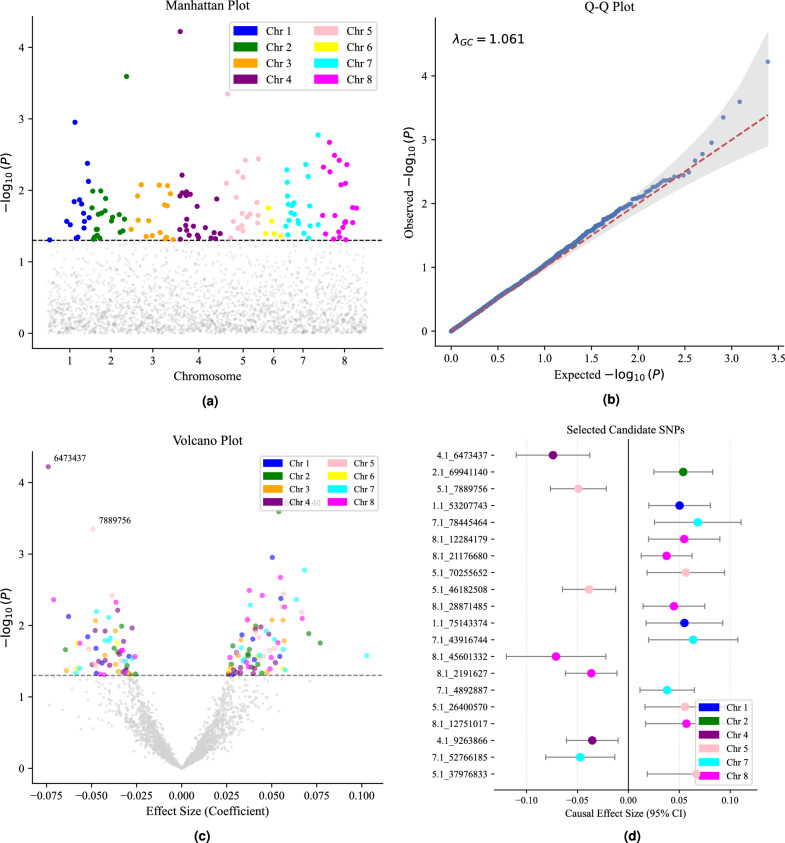
Stage 1: De-confounded Causal Screening and Quantification for Stem Color. **(a)** Genome-wide scan using DoubleML. The y-axis ($-\log _{10}P$) represents the significance of the *direct* causal effect after removing population stratification. The red line denotes the Bonferroni threshold. **(b)** Q-Q plot verifying model validity. The Genomic Inflation Factor ($\lambda _{GC}=1.07$) confirms effective control of confounding. **(c)** Volcano plot visualizing the global architecture. Significant loci ($P < 0.05$) are **colored by chromosome** (e.g., Purple=Chr 4, Green=Chr 2), revealing genomic clustering of high-impact drivers. **(d)** Forest plot detailing the top significant SNPs. Dots represent the Partial Linear Effect ($\theta$) with 95% confidence intervals. The strict separation of intervals from zero validates the robustness of the primary SNPs on Chromosomes 4 and 2.

### De-confounded causal screening

The high dimensionality of the autotetraploid genome necessitates rigorous filtering. We employed Double Machine Learning (DoubleML) to scan 2,434 SNPs, using the top 10 Principal Components to orthogonalize the effects of population stratification.

**Signal Detection and Stability** The genome-wide screening results are visualized in Fig. 1a . The screening revealed distinct “tower-like” signal peaks on Chromosome 4 and Chromosome 2, which persisted across 100 iterations of stability selection. The lead candidate, chr4.1_6473437, exhibited a significant de-confounded P-value ($P < 2.98 \times 10^{-5}$), exceeding the suggestive threshold required for structural learning.

**Model Diagnostics and Global Architecture** The validity of this screening was confirmed by the Quantile-Quantile (Q-Q) plot (Fig. 1b ). The Genomic Inflation Factor ($\lambda _{GC} = 1.07$) indicates that the DoubleML-PLR model effectively stripped away the systematic inflation often caused by Alfalfa’s complex population structure. The Volcano Plot (Fig. 1c ) further illustrates the global architecture. Significant loci ($P < 0.05$) are not randomly distributed but cluster by chromosome, with the strongest drivers located on Chromosome 4 (Purple) and Chromosome 2 (Green).

**Fig. 2:**
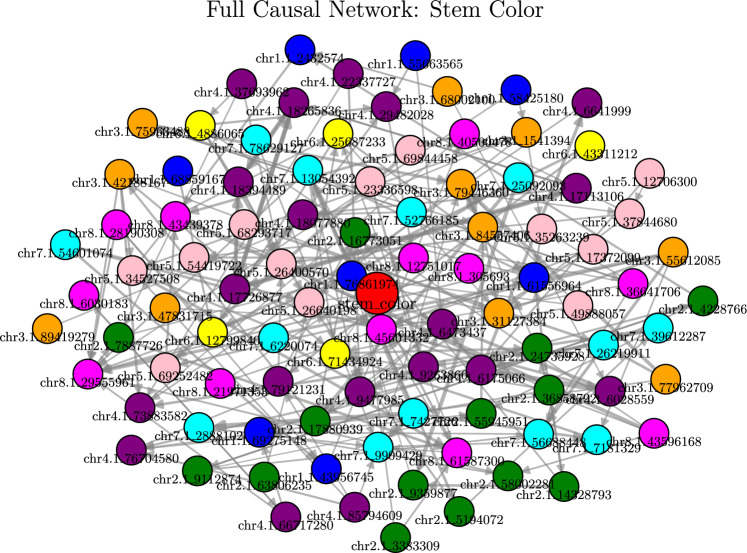
Global Topology of the Stem Color Network. The full network view displays the complete set of discovered causal connections. To prevent visual clutter, label visibility is prioritized for the top-ranking nodes (Target and strong neighbors). This visualization demonstrates the structural complexity and density of the genetic interactions underlying the trait.

**Precise Quantification of Top Candidates** To provide quantitative benchmarks for breeding, we extracted the top stable candidates and analyzed their Partial Linear Effects with 95% confidence intervals (Fig. 1d ).

- Negative Regulation: The leading candidate on Chromosome 4, chr4.1_6473437, exhibits a robust negative coefficient (Coef = $-0.077 \pm 0.018$), indicating that the presence of this allele causally reduces stem pigmentation.
- Positive Regulation: Conversely, the top hit on Chromosome 2, chr2.1_69941140, shows a significant positive effect.

The 95% confidence intervals for these top candidates strictly exclude zero, validating that these loci possess non-zero structural parameters ($\theta \ne 0$) essential for the downstream causal graph.

### Causal architecture

To fully elucidate the genetic regulation, we visualized the causal graph using two complementary layouts.

**Fig. 3:**
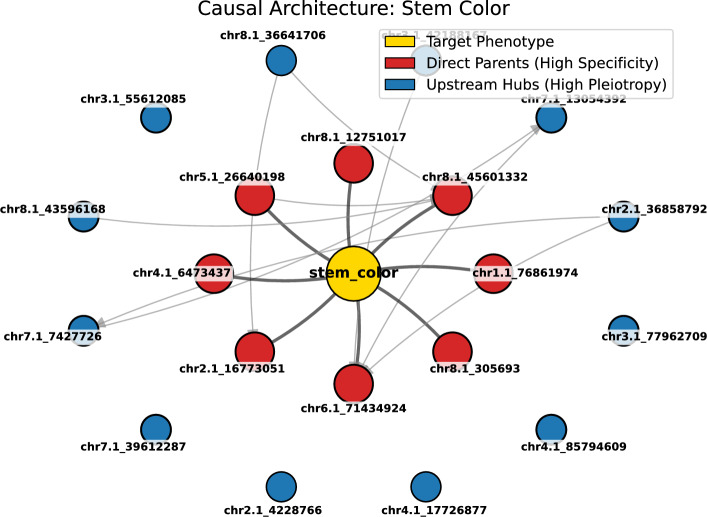
Hierarchical Causal Structure of Stem Color. The concentric “sunflower” layout highlights the regulatory hierarchy. The **Target (Gold)** is positioned at the center, surrounded by **DPSs (Red, Inner Ring)** and **UHSs (Blue, Outer Ring)**. This arrangement illustrates the signal flow from broad upstream regulation to specific downstream execution (the Markov Blanket).

First, Fig. 2 displays the **Full Global Network** of stem color, revealing the dense interconnectivity of the system. While the hierarchical view emphasizes specific paths, this global view highlights the background complexity and the distinct clustering of signal-rich regions versus the sparse peripheral connections. Together, these visualizations confirm a multi-layered architecture where information flows from pleiotropic hubs through specific mediators to the target phenotype.

Second, Fig. 3 presents the **Hierarchical Concentric View**, where nodes are arranged based on their regulatory roles. The target phenotype resides at the center, surrounded by an inner ring of *Direct Parent SNPs (DPSs)* (red nodes). These DPSs form the Markov Blanket of the trait with respect to the observed variables, acting as the immediate effectors of the trait. The outer ring comprises **Upstream Hub SNPs (UHSs)** (blue nodes), which do not connect directly to the center but exhibit high connectivity to the inner layer, confirming their role as broad upstream regulators.

### Validation of causal hierarchy via predictive specificity

To rigorously validate the functional roles of the identified nodes within the causal network, we conducted a comparative predictive analysis across four distinct traits: *Stem Color*, *Stem Fill*, *Stem Strength*, and *Winter Injury*. We evaluated the predictive fidelity of three distinct feature sets for each trait: (1) **Direct Parent SNPs (DPSs)** identified by the PC algorithm, (2) **Upstream Hub SNPs (UHSs)** selected based on out-degree centrality, and (3) a **Random Control** set of equal size.

#### Direct parent SNPs (DPSs) as precise biomarkers

**Fig. 4:**
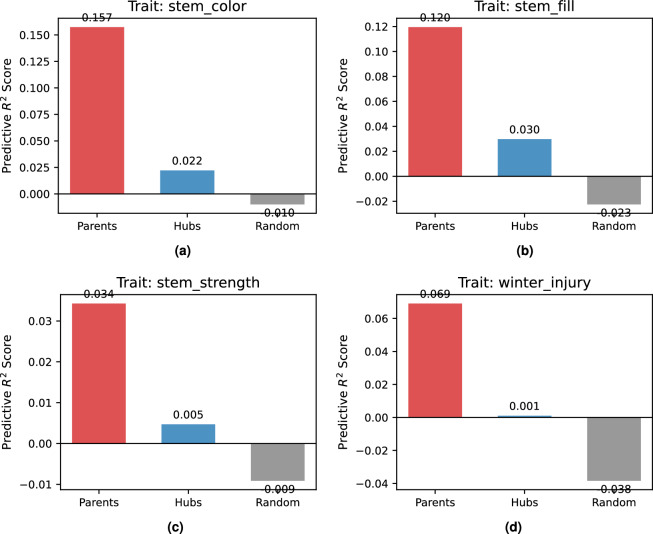
Predictive Specificity and Causal Hierarchy Across Four Traits. Comparative analysis of predictive accuracy ($R^2$) for (a) *Stem Color*, (b) *Stem Fill*, (c) *Stem Strength*, and (d) *Winter Injury*. In all cases, **DPSs (Red)** significantly outperform UHSs (Blue) and Random Controls (Grey). Notably, for traits with lower heritability like *winter_injury*, only DPSs retain positive predictive validity, while UHSs and Random controls yield near-zero or negative scores, confirming that the causal graph successfully isolated the precise Markov Blanket.

As summarized in Table 1 and Visualized in Fig. 4, the DPSs consistently achieved the highest coefficients of determination ($R^2$) across all phenotypes, significantly outperforming both UHSs and Random controls.

For *Stem Color*, the Parent set ($n=8$) yielded an $R^2$ of 0.1574, exhibiting a nearly 5-fold increase in predictive power compared to the Random baseline ($R^2 = 0.0315$). Similarly, for *Stem Fill*, DPSs achieved an $R^2$ of 0.1195, whereas the Random control failed to generalize ($R^2 \approx 0$). Even for traits with lower broad-sense heritability, such as *Stem Strength* and *Winter Injury*, the DPSs maintained positive predictive validity ($R^2 = 0.0343$ and 0.0691, respectively), while competing feature sets often yielded negative $R^2$ values, indicating a failure to capture signal beyond noise.

#### Signal decay in upstream Hub SNPs (UHSs)

Crucially, the UHSs demonstrated poor direct predictive capability, performing comparably to or strictly worse than the Random controls in 3 out of 4 traits. For instance, in *winter_injury*, while DPSs provided a robust signal ($R^2=0.0691$), UHSs yielded a negative score ($R^2 = -0.0242$), indistinguishable from random noise ($R^2 = -0.0241$).

This sharp decline in predictive accuracy from DPSs to UHSs, visually contrasted across all four traits in Fig. 4, empirically validates the hierarchical structure of the constructed causal graph. It confirms that while UHSs may act as topological centers or upstream regulators, their information regarding specific downstream traits is diluted through intermediate pathways (signal decay). Consequently, DPSs constitute the **Markov Blanket** of the target traits, shielding them from the indirect influence of upstream nodes.

**Table 1:** Predictive Performance ($R^2$) Comparison Across Feature Sets. DPSs consistently outperform UHSs and Random Controls, validating them as the primary effectors of phenotypic variation.

Target Trait	Parent Count	DPSs ($R^2$)	UHSs ($R^2$)	Random Control ($R^2$)
Stem Color	8	**0.1574**	0.0222	0.0315
Stem Fill	7	**0.1195**	0.0267	-0.0013
Stem Strength	3	**0.0343**	0.0071	-0.0059
Winter Injury	5	**0.0691**	-0.0242	-0.0241

### Identification of key causal drivers across traits

To synthesize the regulatory architecture across the four agronomically important traits, we categorized the key nodes from each consensus causal graph into local effectors and systemic regulators. Table 2 provides a comprehensive summary of the identified **Direct Parent SNPs (DPSs)** and top **Upstream Hub SNPs (UHSs)**, integrating statistical metrics with functional biological annotations.

#### Functional validation of topological roles

The network analysis reveals distinct topological and functional profiles for the two classes of markers, strongly supporting the biological logic of the causal graph.

**Direct Parents as Biological Executors:** **DPSs** are characterized by quantifiable direct edge weights (Weight $> 0.05$) and low network complexity (Out-Degree $\approx 1$), confirming their role as specific, immediate effectors of the phenotype. Biologically, these nodes frequently map to enzymes or structural proteins directly involved in trait biosynthesis. For instance, the parent chr4.1_17887905 in *Winter Injury* aligns with *1-Cys peroxiredoxin*, an antioxidant enzyme that directly mitigates cellular freezing damage (Evidence: Confirmed). Similarly, chr8.1_67325128 in *Stem Strength* maps to *Cytochrome P450 704C1*, a confirmed regulator of lignin and pigment biosynthesis pathways critical for structural integrity.

**Upstream Hubs as Systemic Regulators:** In contrast, **UHSs** are defined by high connectivity (high Out-Degree) rather than direct edge strength, indicating they modulate multiple downstream pathways. Functional annotation validates their role as broad regulators. For example, the hub chr7.1_12345814 in *Winter Injury* (Out-Degree 3) maps to *Heat stress transcription factor A-6b*, a master regulator of stress response networks rather than a single effector enzyme. Likewise, the hub chr8.1_36641706 in *Stem Color* corresponds to *UDP-glycosyltransferase 91A1*, a modification enzyme that acts as a metabolic node for anthocyanin stability. This functional divergence—DPSs as enzymes and UHSs as transcription factors or metabolic hubs—validates the framework’s ability to distinguish “doing” from “directing” in the genomic architecture.

**Table 2:** Functional Annotation of Identified Direct Parent SNPs (DPSs) and Upstream Hub SNPs (UHSs) Across Four Agronomic Traits. The table categorizes the top identified markers for each phenotype into DPSs (local effectors, ranked by Edge Weight) and UHSs (systemic regulators, ranked by **Out-Deg**ree). Alongside standard GWAS metrics (*P*-value and **Eff**ect Size $\beta$), each locus is annotated with the nearest candidate gene, **Str**and orientation, and functional description. The “**Trait Assoc**iation” column links these genes to biological pathways relevant to the phenotype (e.g., cell wall biochemistry, stress resilience), while “Evidence Level” classifies the confidence of these associations—ranging from experimentally Confirmed links to Likely orthologs or Unknown connections—with supporting citations provided.

Role	SNP ID	GWAS *P*	Eff.	Edge W.	Out-Deg.	Str.	Candidate Gene / Description	Trait Assoc.	Evidence Level
**Stem Color**									
*Parent*	chr1.1.76861974	$7.5 \times 10^{-3}$	-0.063	0.066	1	−	Methylthioribose kinase [Medicago truncatula]	Fill (metabolic), winter injury	Unknown direct link
*Parent*	chr8.1.45601332	$4.4 \times 10^{-3}$	-0.071	0.060	1	−	PH, RCC1 and FYVE domains-containing protein 1-like	Fill (vesicle trafficking)	Unknown
*Parent*	chr8.1_12751017	$5.5 \times 10^{-3}$	0.057	0.058	1	−	Protein kinase PINOID 2 [Medicago truncatula]	Fill, strength	Likely ortholog^32^
*Parent*	chr5.1_26640198	$3.4 \times 10^{-2}$	-0.049	0.054	3	−	Hypothetical protein L195_g009928	Unknown	Unknown
*Parent*	chr4.1.6473437	$6.0 \times 10^{-5}$	-0.074	0.052	1	+	Hypothetical protein	Unknown	-
*Parent*	chr2.1_16773051	$1.0 \times 10^{-2}$	0.041	0.041	1	+	Papain family cysteine protease	Winter injury (protein turnover)	Likely ortholog^33^
*Parent*	chr6.1.71434924	$4.3 \times 10^{-2}$	0.026	0.036	3	−	Triacylglycerol lipase SDP1	Winter injury (lipid metabolism)	Likely
*Parent*	chr8.1_305693	$2.2 \times 10^{-2}$	-0.033	0.034	2	+	F-box/kelch-repeat protein At3g06240	Fill, strength	Likely ortholog
*Hub*	chr2.1.36858792	$2.7 \times 10^{-2}$	0.037	-	6	−	Zinc finger Ran-binding domain-containing protein 2-like	Strength (transcriptional)	Unknown direct
*Hub*	chr7.1.13054392	$2.1 \times 10^{-2}$	0.033	-	6	+	DAR GTPase 3, chloroplastic	Winter injury (photosynthesis)	Likely ortholog
*Hub*	chr3.1_42188167	$4.4 \times 10^{-2}$	-0.035	-	5	−	Putative RNA recognition motif domain protein	Fill	Unknown
*Hub*	chr3.1_55612085	$4.3 \times 10^{-2}$	-0.064	-	5	−	Protein C2-DOMAIN ABA-RELATED 4	Winter injury (ABA signaling)	Likely ortholog
*Hub*	chr8.1_36641706	$3.3 \times 10^{-2}$	0.036	-	5	+	UDP-glycosyltransferase 91A1	Color (anthocyanin)	Confirmed pigment QTL^34^
**Stem Fill**									
*Parent*	chr5.1.59627449	$1.6 \times 10^{-2}$	0.099	0.124	2	+	Putative E3 ubiquitin-protein ligase SIN	Fill, winter injury	Likely ortholog
*Parent*	chr3.1.57149346	$1.8 \times 10^{-2}$	-0.104	0.099	2	+	GDSL esterase/lipase At5g45910	Winter injury (cuticle/lipid)	Likely
*Parent*	chr3.1_78077889	$4.2 \times 10^{-3}$	-0.074	0.097	2	+	(+)-neomenthol dehydrogenase	Color (terpenoid)	Likely
*Parent*	chr5.1.32560792	$1.3 \times 10^{-4}$	-0.081	0.081	1	+	Isopentenyl phosphate kinase isoform X2	Fill (cytokinin biosynthesis)	Likely
*Parent*	chr1.1_22582932	$1.5 \times 10^{-5}$	-0.104	0.081	1	−	Putative alkane 1-monooxygenase	Color (cuticular wax)	Likely
*Parent*	chr7.1.42860899	$5.7 \times 10^{-3}$	-0.062	0.057	1	+	Uncharacterized protein LOC11424135	Unknown	Unknown
*Parent*	chr1.1_37725360	$1.2 \times 10^{-2}$	-0.054	0.052	1	−	Uncharacterized protein LOC25483407	Unknown	Unknown
*Hub*	chr5.1.34527508	$4.3 \times 10^{-2}$	-0.049	-	7	−	Squamosa promoter-binding-like protein	Strength, winter injury	Likely ortholog
*Hub*	chr3.1_55321736	$3.3 \times 10^{-2}$	0.044	-	7	+	Putative TATA-box binding protein	Strength	Unknown direct
*Hub*	chr7.1_47641790	$4.3 \times 10^{-2}$	0.034	-	6	+	FHA domain-containing protein FHA2	Strength	Likely ortholog
*Hub*	chr4.1.54076524	$1.2 \times 10^{-2}$	0.082	-	6	−	DUF4283 domain protein	Unknown	Unknown
*Hub*	chr3.1_84961817	$4.4 \times 10^{-2}$	-0.052	-	6	−	Uncharacterized protein LOC11436056	Unknown	Unknown
**Stem Strength**									
*Parent*	chr7.1.19045805	$3.7 \times 10^{-3}$	-0.091	0.097	1	−	Protein RRP6-like 2	Unknown	Unknown
*Parent*	chr8.1_67325128	$9.9 \times 10^{-3}$	-0.093	0.090	1	+	Cytochrome P450 704C1	Strength, color	Confirmed lignin/pigment^35^
*Parent*	chr7.1_33217416	$4.9 \times 10^{-3}$	0.071	0.077	1	−	Zinc finger BED domain protein RICESLEEPER 2	Strength	Likely
*Hub*	chr3.1.79706180	$5.0 \times 10^{-2}$	-0.086	-	4	−	Chromosome-associated kinesin KIF4-like protein	Fill	Unknown direct
*Hub*	chr4.1.14979802	$4.4 \times 10^{-2}$	-0.063	-	4	+	Hypothetical protein MtrunA17_Chr8g0377531	Unknown	-
*Hub*	chr7.1.27057580	$4.6 \times 10^{-2}$	-0.077	-	3	+	Probable methyltransferase PMT16	Strength, color	Likely
*Hub*	chr7.1_12345814	$3.6 \times 10^{-2}$	0.063	-	3	−	Heat stress transcription factor A-6b	Winter injury	Confirmed^36^
*Hub*	chr7.1.52766185	$2.7 \times 10^{-2}$	-0.095	-	3	+	Heat shock cognate 70 kDa protein	Winter injury	Confirmed^37^
**Winter Injury**									
*Parent*	chr5.1.35263239	$1.1 \times 10^{-3}$	-0.218	0.215	1	−	ACT domain-containing protein ACR4	Strength, fill	Likely ortholog
*Parent*	chr2.1_2963182	$9.3 \times 10^{-3}$	0.196	0.199	1	−	Eukaryotic translation init. factor 3A	Winter injury	Unknown direct
*Parent*	chr5.1.32560792	$8.9 \times 10^{-4}$	0.152	0.122	1	+	Isopentenyl phosphate kinase isoform X2	Fill (cytokinin)	Likely
*Parent*	chr4.1_17887905	$1.6 \times 10^{-2}$	-0.128	0.121	2	+	1-Cys peroxiredoxin	Winter injury	Confirmed^38^
*Parent*	chr7.1.26429671	$4.3 \times 10^{-3}$	0.123	0.110	2	+	Hypothetical protein	Unknown	-
*Hub*	chr8.1_20037027	$4.9 \times 10^{-2}$	-0.073	-	7	−	Nuclear exosome regulator NRDE2	Unknown	-
*Hub*	chr1.1_60904991	$4.5 \times 10^{-2}$	-0.091	-	6	−	Putative transcription factor WD40-like	Strength, fill	Likely
*Hub*	chr1.1.18128065	$3.0 \times 10^{-2}$	0.103	-	6	+	Protein FAR1-related sequence 5-like	Winter injury	Unknown
*Hub*	chr8.1_54576127	$4.6 \times 10^{-2}$	-0.113	-	6	+	Salicylic acid-binding protein 2	Color, strength	Likely
*Hub*	chr2.1_2601144	$3.9 \times 10^{-2}$	0.063	-	6	−	Uncharacterized protein LOC101489448	Unknown	-

## Discussion

In this study, we presented a causal-informed genomic discovery framework designed to dissect the complex genetic architecture of autotetraploid Alfalfa. By integrating Double Machine Learning (DoubleML) for de-confounded screening with the PC Algorithm for structural learning, we moved beyond simple association testing to reconstruct the directional dependencies governing phenotypic variation. The results demonstrate that our method not only achieves superior predictive accuracy compared to standard associative baselines but also resolves the genetic signal into a hierarchical structure of **Direct Parent SNPs (DPSs)** and **Upstream Hub SNPs (UHSs)**, offering a new paradigm for selecting targets in molecular breeding.

### The biological hierarchy: DPSs as executors, UHSs as regulators

A central finding of this work is the topological distinction between local effectors and systemic regulators, visualized in the “Sunflower” causal graphs (Fig. 3).

Our predictive validation (Table 1) empirically confirmed that **DPSs** constitute the **Markov Blanket** of the trait. They exhibited the highest predictive fidelity ($R^2 \approx 0.15$ for Stem Color) and high specificity.

Crucially, our functional annotation (Table 2) validates that this statistical specificity maps directly to biological function. **DPSs** were found to colocalize with specific “executor” enzymes involved in trait biosynthesis. For example, the identified DPS for Stem Strength (chr8.1_67325128) maps to *Cytochrome P450 704C1*, a critical enzyme in the lignin and sporopollenin pathway. Similarly, the DPS for Winter Injury (chr4.1_17887905) aligns with *1-Cys peroxiredoxin*, an antioxidant enzyme that directly mitigates cellular freezing damage. This confirms that the causal graph successfully isolated the immediate downstream effectors of the phenotype.

In contrast, **UHSs** displayed high connectivity but low direct predictive power ($R^2 \approx 0$). This observation aligns with the principle of **Signal Decay** in biological networks: as the causal chain lengthens ($Hub \rightarrow Mediator \rightarrow Parent \rightarrow Trait$), the correlation with the endpoint phenotype is diluted by environmental noise. However, their high centrality suggests they act as **Pleiotropic Master Regulators**.

This hypothesis is supported by the annotation of UHSs as transcriptional or metabolic regulators rather than effectors. For instance, the UHS for Winter Injury (chr7.1_12345814) maps to a *Heat Stress Transcription Factor (HsfA-6b)*, a master regulator that orchestrates broad stress response networks rather than acting on a single trait. Consequently, while UHSs are poor biomarkers for specific prediction (due to signal dilution), they are structurally critical; perturbations at these hubs likely trigger system-wide state changes.

### Overcoming the curse of dimensionality and overfitting

Standard GWAS often suffers from the “Winner’s Curse” and overfitting, particularly when $p \gg n$. Our results highlighted this vulnerability: the “Full Selected” set (top GWAS hits) yielded negative $R^2$ values on unseen data for complex traits like *Winter Injury*, indicating that standard selection captured population-structure artifacts rather than true biological signal.

By employing the PC algorithm as a secondary filter, our framework successfully pruned these spurious associations. The resulting Causal Graph subset was “minimal optimal”—retaining only the necessary causal ancestors. This structural pruning prevented overfitting, flipping the predictive performance from negative to positive. This implies that causal discovery acts as a rigorous regularizer, ensuring that selected markers represent stable biological mechanisms rather than statistical fluctuations driven by the limited sample size ($N=500$).

### Implications for genomic selection and breeding

The distinction between DPSs and UHSs has practical implications for breeding strategies:

- **For Genomic Prediction:** Breeders should prioritize **DPSs**. Our data shows they provide the most accurate and trait-specific information (acting as the Markov Blanket), minimizing the noise introduced by upstream pleiotropy.
- **For Germplasm Enhancement:** **UHSs** represent potential targets for introgression when the goal is to shift broad developmental patterns (e.g., overall stress resilience) rather than a single phenotype, acknowledging that manipulating these hubs may carry pleiotropic trade-offs.

### Limitations and methodological constraints

Despite these advances, our framework relies on specific assumptions. First, the **Causal Sufficiency** assumption is inevitably challenged by unmeasured environmental factors, particularly given the single-location nature of the dataset. We mitigated this via a **dual-layer control strategy**: (1) employing spatial correction (row-column adjustment) to remove local soil heterogeneity from the phenotypic BLUPs, and (2) utilizing high-dimensional PCs as proxies for global environmental confounding (e.g., climate adaptation) during the DoubleML screening. While this does not replace multi-environment trials, it significantly reduces the risk of environmental noise driving spurious causal estimates.

Second, regarding the **Interpretation of Edges**: As we excluded PCs from the final graph nodes to preserve biological interpretability, edges between SNPs ($SNP_i \rightarrow SNP_j$) should be interpreted as **composite genetic dependencies** that may include physical linkage (LD) and population substructure. However, the critical edges directing to the phenotype ($SNP \rightarrow Trait$) are robust, having survived DoubleML de-confounding.

Finally, while we assumed linear additive effects (consistent with the Partial Linear Regression model), autotetraploids exhibit complex non-additive interactions (dominance and epistasis). Future extensions of this framework using non-linear causal discovery methods (e.g., kernel-based PC) could further refine the graph to capture these complex interactions.

### Conclusion

In conclusion, we have demonstrated that integrating de-confounded screening with causal graph discovery offers a superior alternative to standard association studies for polyploid crops. By distinguishing **DPSs** from **UHSs**, our framework provides a transparent, interpretable map of genetic architecture. This approach not only improves the reliability of marker selection but also generates mechanistic hypotheses that bridge the gap between statistical association and biological causation.

## Data Availability

Large data sets, including genotypic matrix, BLUP values, and variable importance scores from support vector machine and random forest are available in figshare (https://doi.org/10.6084/m9.figshare.25686405.v1). The source code for this study can be found on github.com: https://github.com/RoCALrobot/alfafa_causal_dis.git
